# Antibody targeting facilitates effective intratumoral siRNA nanoparticle delivery to HER2-overexpressing cancer cells

**DOI:** 10.18632/oncotarget.7076

**Published:** 2016-01-30

**Authors:** Maria C. Palanca-Wessels, Garrett C. Booth, Anthony J. Convertine, Brittany B. Lundy, Geoffrey Y. Berguig, Michael F. Press, Patrick S. Stayton, Oliver W. Press

**Affiliations:** ^1^ Clinical Research Division and Center for Intracellular Delivery of Biologics, Fred Hutchinson Cancer Research Center, Seattle, WA, USA; ^2^ Department of Medicine, Hematology Division, University of Washington, Seattle, WA, USA; ^3^ Department of Bioengineering and Center for Intracellular Delivery of Biologics, University of Washington, Seattle, WA, USA; ^4^ Department of Pathology, University of Southern California, Los Angeles, CA, USA

**Keywords:** HER2 antibody, siRNA, targeted antibody delivery, ovarian cancer, breast cancer

## Abstract

The therapeutic potential of RNA interference (RNAi) has been limited by inefficient delivery of short interfering RNA (siRNA). Tumor-specific recognition can be effectively achieved by antibodies directed against highly expressed cancer cell surface receptors. We investigated the utility of linking an internalizing streptavidin-conjugated HER2 antibody to an endosome-disruptive biotinylated polymeric nanocarrier to improve the functional cytoplasmic delivery of siRNA in breast and ovarian cancer cells *in vitro* and in an intraperitoneal ovarian cancer xenograft model *in vivo*, yielding an 80% reduction of target mRNA and protein levels with sustained repression for at least 96 hours. RNAi-mediated site specific cleavage of target mRNA was demonstrated using the 5′ RLM-RACE (RNA ligase mediated-rapid amplification of cDNA ends) assay. Mice bearing intraperitoneal human ovarian tumor xenografts demonstrated increased tumor accumulation of Cy5.5 fluorescently labeled siRNA and 70% target gene suppression after treatment with HER2 antibody-directed siRNA nanocarriers. Detection of the expected mRNA cleavage product by 5′ RLM-RACE assay confirmed that suppression occurs via the expected RNAi pathway. Delivery of siRNA via antibody-directed endosomolytic nanoparticles may be a promising strategy for cancer therapy.

## INTRODUCTION

The discovery of the RNA interference (RNAi) mechanism over a decade ago by Andrew Fire and Craig Mello stimulated intense interest in the potential application of short interfering RNA (siRNA), not only as a useful tool for the mechanistic study of cellular pathways but more significantly as a novel disease therapeutic. The ensuing years have resulted in a flurry of preclinical investigations and early phase clinical trials designed to test the utility of siRNA for the treatment of a variety of human diseases. [1, 2] In particular, siRNA for suppression of genes critical for tumor growth or resistance to chemotherapy may improve clinical outcomes and patient survival. The demonstration of the RNAi-mediated mechanism of target mRNA cleavage in human tumors from patients treated in Phase I clinical trials provides hope for clinical use of siRNA, however, the major obstacle of cell specific delivery remains to be overcome. [3, 4]

The selective recognition of tumors by antibodies has been harnessed to directly elicit apoptosis, stimulate the immune-mediated clearance of cancer cells, or target cytotoxic agents to cancer cells. HER2 (also known as ERBB2 or NEU) is an internalizing cell surface receptor overexpressed or mutated in a variety of solid tumors including breast, gastric, lung and ovarian cancers. [5–9] Treatment with trastuzumab, a humanized mouse antibody directed against HER2, has become the standard of care for HER2-overexpressing breast cancer [6] and HER2-overexpressing metastatic gastric cancer. [10] HER2 has additionally been investigated as a portal through which receptor targeted antibodies or aptamers can deliver peptides, [11–13] siRNA [14, 15] or small molecule drugs [16] into cells. Recently, a drug conjugated form of trastuzumab (ado-trastuzumab emtansine) that facilitates the intracellular delivery of the cytotoxic microtubule inhibiting agent mertansine was approved for the treatment of metastatic breast cancer. [17] Trastuzumab in combination with chemotherapy improved outcomes in gastric cancer patients, but single agent trastuzumab in ovarian cancer patients did not extend survival, although patients were selected for the clinical trial using relatively insensitive immunohistochemical assays without any confirmation of *HER2* gene amplification status. [10, 18]

We previously developed a modular siRNA delivery system using a biotinylated endosome disrupting polymer that permits facile testing of combinations of antibody and siRNA and can be potentially tailored to various tumor types. [19] We hypothesized that a trastuzumab-directed siRNA nanoparticle could be used to enhance target gene suppression in HER2-overexpressing ovarian cancer cells. Ovarian cancer remains the most deadly cancer in women primarily due to its advanced state at diagnosis and rapid development of drug resistance. Little progress has been made over the past 20 years in improving the overall survival of patients, approximately 40% at 5 years, underscoring the need for novel therapeutic agents. [20]

**Figure 1:**
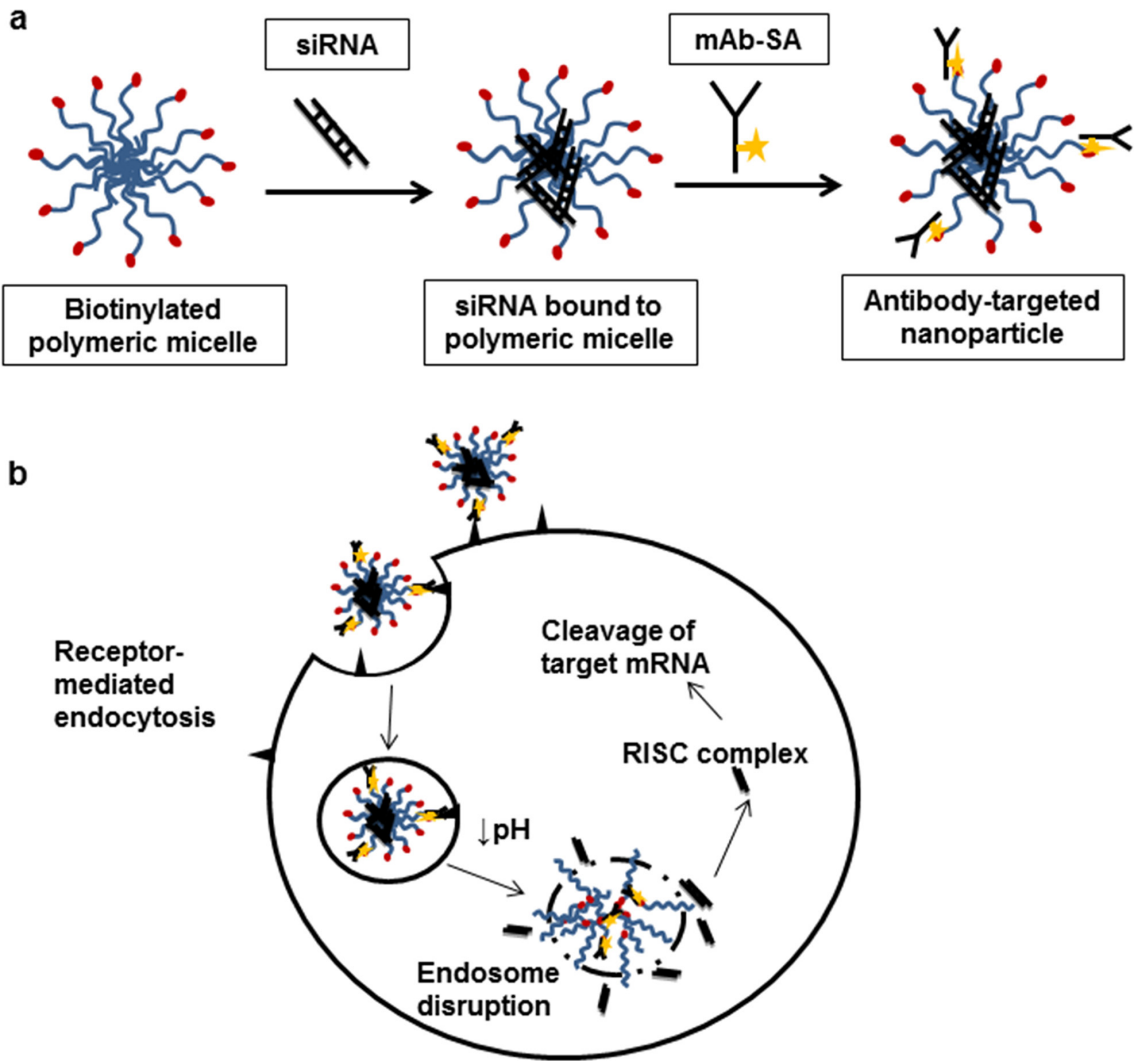
Antibody-targeted nanoparticle formation and intracellular siRNA delivery **a.** The biotinylated diblock copolymer carrier consists of two modules: (1) a biotinylated cationic block comprised of poly(DMAEMA) and (2) an amphiphilic block consisting of DMAEMA, BMA and PPA groups. The linear polymer chains spontaneously form polymeric micelles with biotin exposed on the surface of the micelles. Polyplexes are formed by adding siRNA which associate with micelles via electrostatic interactions. Antibody-streptavidin conjugates (mAb-SA) are added and attach to available biotin moieties on the polymeric micelle surface resulting in antibody-targeted nanoparticles. **b.** Intracellular delivery is initiated by the recognition and binding of the antibody targeted nanoparticle to the cognate internalizing cell surface antigen on the surface of the tumor cell. Receptor-mediated endocytosis is triggered and the nanoparticle is internalized into the cell. As endosomal maturation occurs, the pH in the endosomal compartment drops and carboxyl groups in the amphilic block are protonated causing a conformational change of the polymer. This exposes the hydrophobic BMA side groups and disrupts the endosomal membrane leading to release of siRNA. siRNA is incorporated into the cytoplasmic RISC complex leading to catalytic cleavage of target mRNA and reduction of gene expression.

Our group previously demonstrated the effectiveness of siRNA delivery to cells *in vitro* via a pH-sensitive endosomolytic diblock co-polymer carrier bearing an internalizing antibody directed against the CD22 receptor expressed on lymphoma cells. [19] The linear carrier consists of: 1) a pH-responsive ampholyte block of poly(DMAEMA) (dimethylaminoethyl methacrylate), BMA (butylmethacrylate), and PAA (propylacrylic acid) groups; 2) a cationic poly(DMAEMA) block for binding siRNA; and 3) a terminal biotin to enable linkage to a streptavidin-conjugated monoclonal antibody (mAb-SA). Electrostatic interactions promote complexation of siRNA to the polymeric micelles (Figure 1a). Targeted nanoparticles are formed by subsequent addition of mAb-SA which attach to exposed surface biotin on micelles. Binding of antibody to cognate antigen stimulates receptor-mediated endocytosis and uptake into the tumor cell (Figure 1b). Subsequent protonation of PAA in the acidic environment of late endosomes induces a conformational change to predominantly hydrophobic unimers, disrupting the endosomal membrane and releasing siRNA into the cytoplasm. The modularity of this system permits testing of combinations of antibodies and siRNA customized to different tumor types. We sought to demonstrate the versatility and effectiveness of our polymer carrier system both *in vitro* and *in vivo* using the HER2 antibody, trastuzumab, in a solid tumor xenograft model of ovarian cancer.

## RESULTS

### Intracellular uptake of nanoparticles by HER2-overexpressing cancer cells

**Figure 2:**
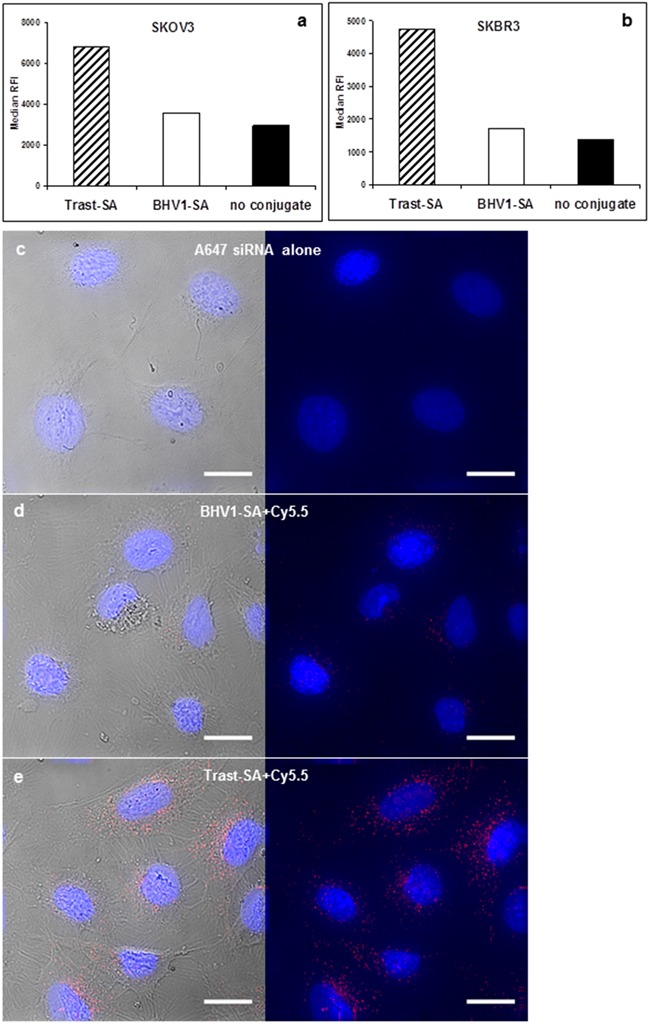
HER2 antibody conjugate Trast-SA enhances the uptake of siRNA-containing nanoparticles into HER2-overexpressing SKOV3 ovarian and SKBR3 breast cancer cells **a.** SKOV3 and **b.** SKBR3 cells were treated continuously for 1 hour with Trast-SA or BHV1-SA bearing nanoparticles or naked nanoparticles containing AlexaFluor 647 (AF647) labeled siRNA, trypsinized, then analyzed by flow cytometry for median relative fluorescence intensity (RFI). Representative results of 2 separate experiments are shown. **c-e.** SKOV3 cells grown on chambered glass slides were treated for 2 hours with (c) fluorescent AF647 labeled siRNA alone or contained within (d) BHV1-SA or (e) Trast-SA bearing nanoparticles prior to being rinsed and incubated with medium without nanoparticles. After 24 hours, cells were fixed, counterstained with mounting medium containing DAPI DNA stain, then visualized by fluorescence microscopy. Images were taken with a DeltaVision Elite wide deconvolution microscope at the original magnification of 80X. For each panel (c-e), both the fluorescence image (right) and fluorescence image merged with its respective differential interference contrast image (left) are shown. Scale bar equals 20 microns.

Binding and internalization of the trastuzumab-polymer siRNA nanoparticle was confirmed by flow cytometry in both HER2-overexpressing SKOV3 ovarian cancer and SKBR3 breast cancer cells using fluorescent AlexaFluor 647 labelled siRNA (Figure 2). Fluorescence intensity after 1 hour of incubation was markedly higher in cells exposed to nanoparticles targeted with streptavidin-conjugated trastuzumab (Trast-SA) compared to non-targeted bovine herpes virus-1 antibody conjugate (BHV1-SA) or naked nanoparticles. Confocal microscopy of SKOV3 cells 24 hours after treatment with AlexaFluor 647 labeled siRNA showed a punctate pattern of fluorescence consistent with endocytic uptake (Figure 2). Enhanced uptake was similarly observed in SKOV3 cells using a different HER2 antibody 10H8 which recognizes a separate epitope on the HER2 receptor (Supplementary Figure S1a).

### RNAi-mediated suppression via HER2 antibody-linked siRNA carrier

**Figure 3:**
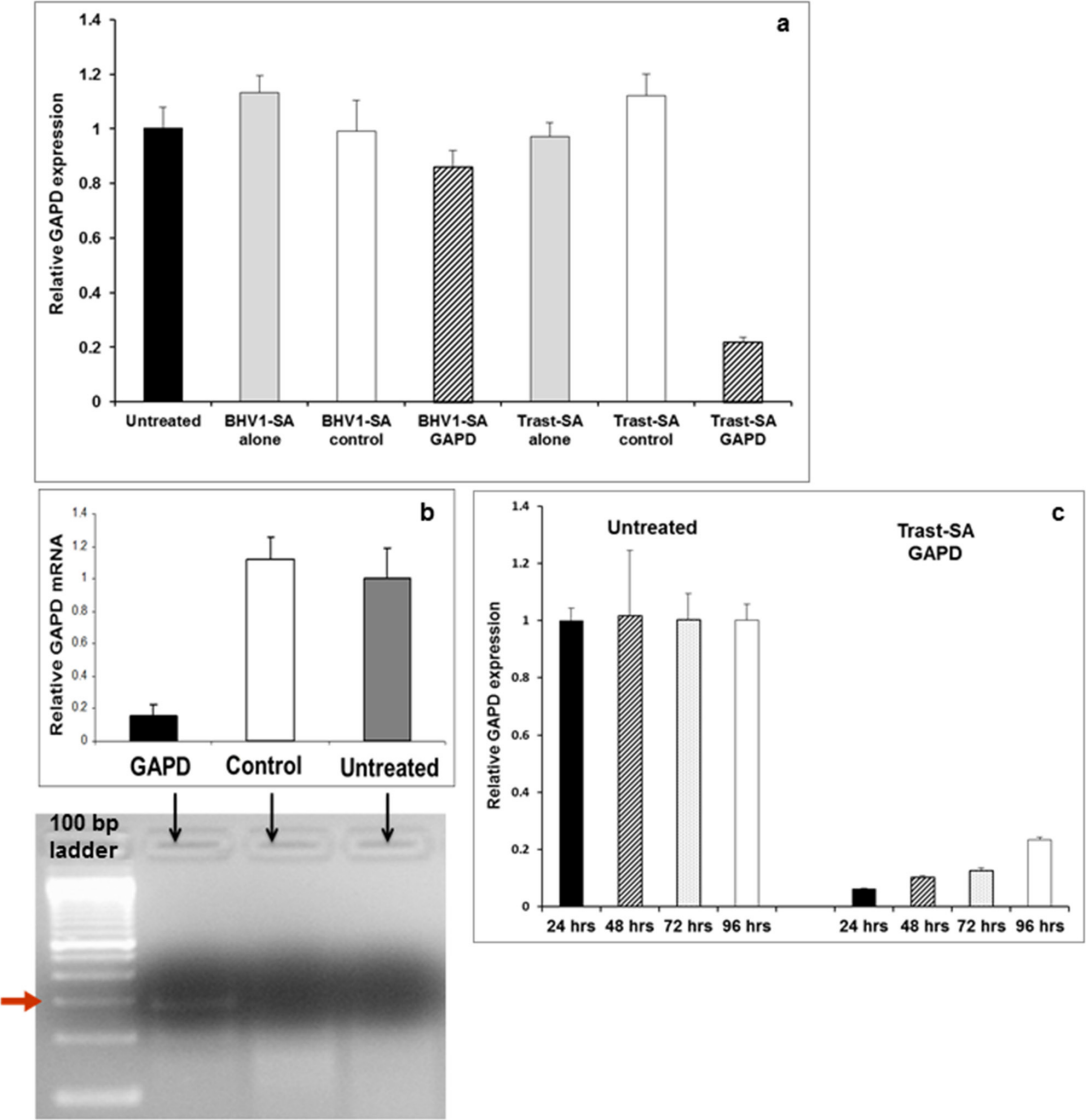
Suppression of GAPD gene expression by Trast-SA polymer mediated siRNA delivery in SKOV3 ovarian cancer cells **a.** SKOV3 cells were incubated for 2 hours in triplicate wells with *GAPD* or negative control siRNA contained in nanoparticles bearing Trast-SA or BHV1-SA conjugates or the respective antibody conjugates alone. Medium containing nanoparticles was then replaced with fresh medium alone and cells subsequently harvested 48 hours after the initial treatment and RNA extracted for qRT-PCR. Results are representative of three separate experiments. **b.** Verification of mRNA suppression via an RNAi mechanism was performed on SKOV3 cells that were pulse treated for 2 hours with Trast-SA bearing nanoparticles containing either *GAPD* or negative control siRNA and RNA extracted 48 hours later for qRT-PCR and 5′RLM-RACE analysis. The bar graph demonstrates reduction of target GAPD mRNA levels as measured by qRT-PCR in cells treated with GAPD but not control siRNA. Below the graph, gel electrophoresis of 5′ RLM-RACE assay products from the respective treatments showed the predicted 281 base pair *GAPD* mRNA cleavage product only in the Trast-SA *GAPD* siRNA treated but not control siRNA or untreated cells supporting an RNAi mechanism for reduced GAPD expression. **c.** Sustained suppression of *GAPD* expression was observed in SKOV3 EA8 luciferase expressing cells treated for 2 hours with Trast-SA containing *GAPD* siRNA then analyzed for *GAPD* mRNA level via qRT-PCR at various timepoints after treatment.

Functional delivery was assessed in both HER2-overexpressing ovarian and breast cancer cells using siRNA directed against the ubiquitously expressed glyceraldehyde-3-phosphate dehydrogenase (*GAPD*) enzyme gene. Two hour pulse treatment of SKOV3 ovarian cancer cells with Trast-SA targeted nanoparticles containing *GAPD* siRNA resulted in greater reduction of *GAPD* expression at 48 hours compared to BHV1-SA as assessed by quantitative RT-PCR (Figure 3a) and GAPD enzyme activity (Supplementary Figure S1b). Verification of the RNAi mechanism of gene suppression was accomplished by the detection of the predicted 281 base pair fragment of *GAPD* mRNA using the 5′ RLM-RACE assay (RNA ligase-mediated rapid amplification of cDNA ends) (Figure 3b). Sequencing of the isolated fragment verified that cleavage occurred at the expected site in the *GAPD* mRNA. Robust suppression of the *GAPD* gene was demonstrated for at least 96 hours after 2 hour pulse treatment of luciferase-expressing SKOV3 EA8 cells (Figure 3c). *GAPD* gene suppression was similarly demonstrated in the HER2-overexpressing breast cancer cell lines SKBR3 and BT-474 (Supplementary Figure S2). Treatment of cells with nanoparticles did not elicit cytotoxicity nor induce TLR-3 (toll-like receptor 3) activated immune response genes *STAT1* (signal transducer and activator of transcription-1) or *OAS1* (2′-5′ oligoadenylate synthetase-1) genes (Supplementary Figure S3).

**Figure 4:**
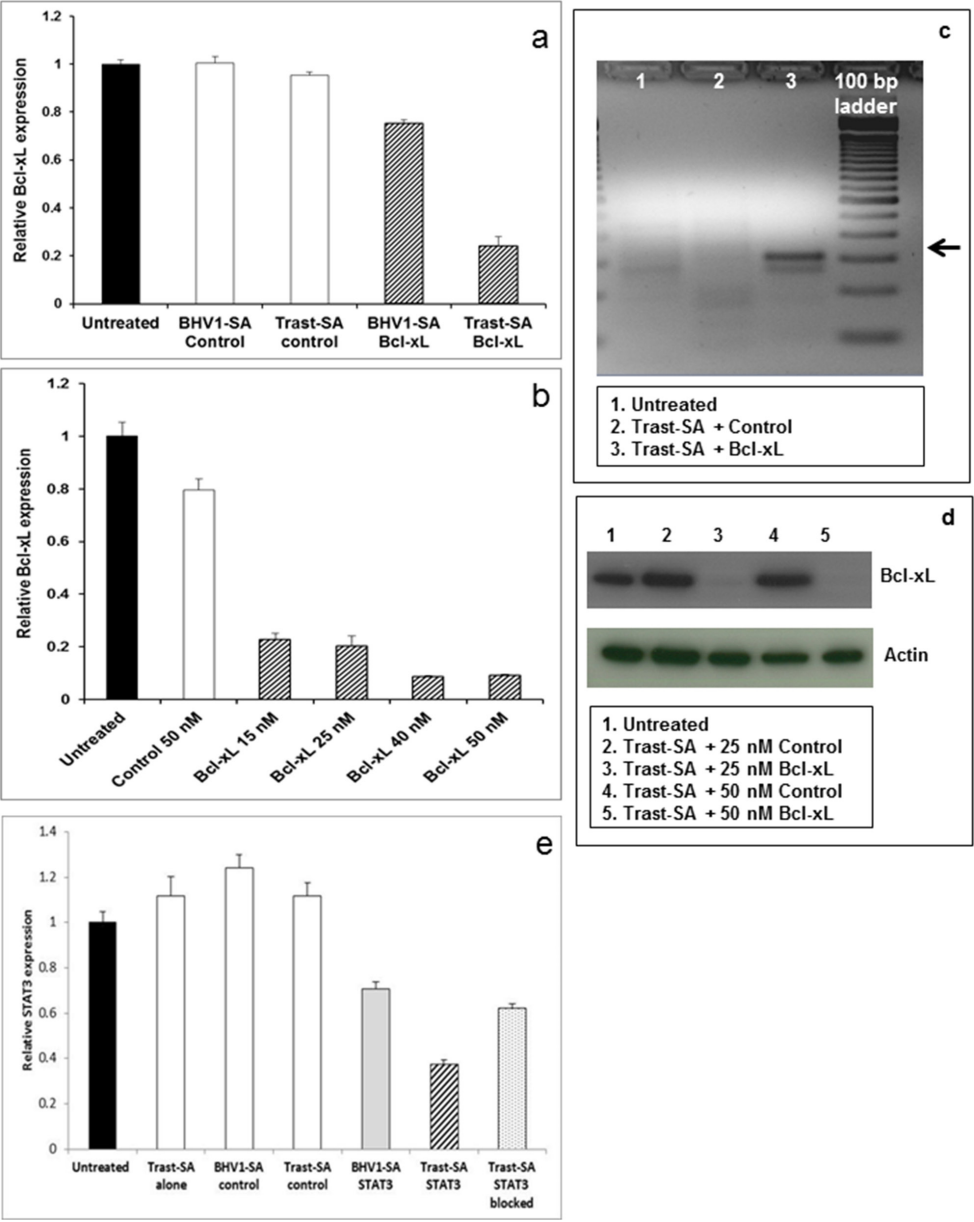
Effective suppression of oncogenes Bcl-xL and STAT3 in cancer cells SKOV3 EA8 cells were treated for 2 hours with nanoparticles bearing Trast-SA or BHV1-SA conjugates and containing 25 nM siRNA directed against *BCL-XL* or a scrambled negative control bearing no homology to any known gene sequence. Cells were rinsed to remove medium containing nanoparticles and fresh medium introduced. After 48 hours, cells were harvested and RNA collected for **a.** qRT-PCR analysis and **c.** detection of the expected 309 base pair *BCL-XL*mRNA fragment (red arrow) by the 5′RLM-RACE assay. SKOV3 EA8 cells were similarly treated for 2 hours with Trast-SA targeted nanoparticles at a dose range (15 to 50 nM) of *BCL-XL* siRNA or negative control siRNA at 25 or 50 nM. RNA or protein was collected at 48 hours for **b.** qRT-PCR analysis and **d.** Western blot respectively. Results from cells treated with 25 nM or 50 nM negative control siRNA are shown since *BCL-XL* expression levels did not vary by negative control siRNA dose. HER2 receptors on SKOV3 EA8 cells were blocked by incubation with 37.5 nM of Trast-SA alone for 30 minutes prior to 2 hour incubation with Trast-SA targeted polyplexes containing 25 nM siRNA directed against *STAT3*. Other cells were treated with nanoparticles containing negative control or *STAT3* siRNA bearing the indicated antibody conjugate targeting without preincubation. RNA was harvested 48 hours later and *STAT3* levels assessed by qRT-PCR **e.** All qRT-PCR analyses were performed for triplicate samples with standard deviation shown. Similar results were observed in duplicate experiments.

In order to demonstrate that suppression could be achieved using different clinically relevant siRNA sequences, we tested the functional delivery of siRNA designed against genes associated with chemotherapy resistance. High levels of *BCL-XL* (B-cell lymphoma-extra large, also known as *BCL2L1* or BCL2-like-1) are found in platinum resistant ovarian cancer cells and inhibition of *BCL-XL* confers sensitivity to chemotherapeutic agents. [21, 22] *STAT3* (signal transducer and activator of transcription-3) has been shown to upregulate *BCL-XL* in solid tumors. [23] Significant suppression of *BCL-XL* was demonstrated in SKOV3 EA8 cells pulse treated for 2 hours with Trast-SA targeted nanoparticles containing *BCL-XL* siRNA (Figure 4a) and showed an expected dose response with increasing siRNA concentrations (Figure 4b). The predicted 309 base pair mRNA cleavage product was demonstrated by the 5′RLM-RACE assay in cells treated with *BCL-XL* siRNA but not negative control siRNA (Figure 4c). The mRNA cleavage product identity was verified by sequencing the cDNA band purified from the gel. Reduction of BCL-XL protein in cells was demonstrated by Western blot analysis (Figure 4d). Similar results were shown using a second *BCL-XL* siRNA sequence (data not shown). Reduction of *STAT3* mRNA was shown with qRT-PCR. Pretreatment with Trast-SA conjugate blocked HER2-mediated uptake of Trast-SA bearing nanoparticles containing *STAT3* siRNA as demonstrated by reduction of *STAT3* gene suppression to a level comparable to that achieved with non-targeting BHV1-SA conjugate (Figure 4e).

### Localization of siRNA to intraperitoneal ovarian tumor with HER2 antibody

**Figure 5:**
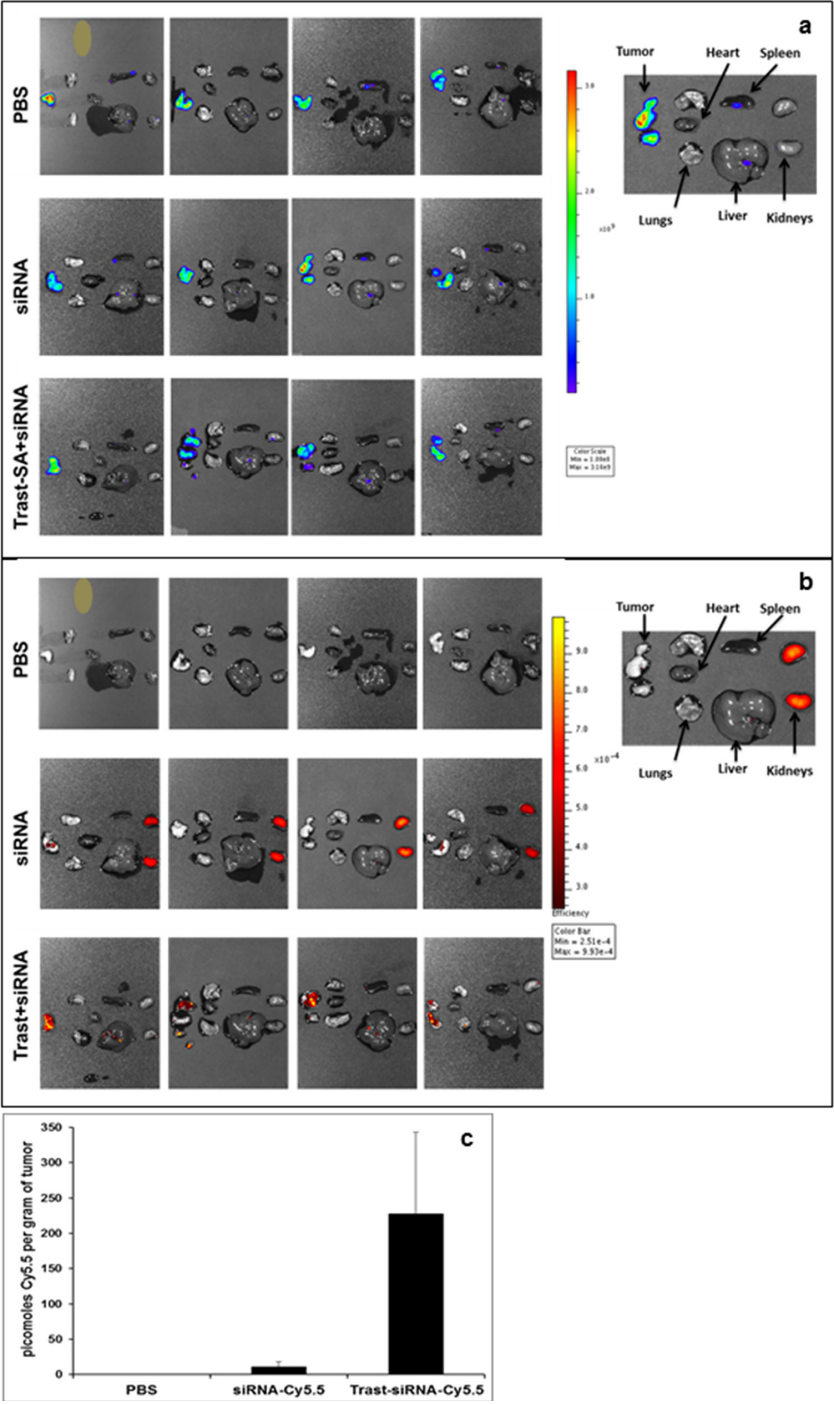
Intraperitoneal ovarian tumor localization of Cy5.5 fluorescently labeled siRNA after administration of HER2 targeted carrier Athymic mice were injected intraperitoneally with SKOV3 EA8 luciferase expressing cells and tumor establishment verified by bioluminescence imaging using the Xenogen IVIS system. Two weeks after tumor cell injection, 4 mice per treatment group were injected with phosphate buffered saline (PBS), 2 mg/kg Cy 5.5 labelled siRNA alone or contained within Trast-SA targeted nanoparticles. Twenty-four hours later, mice were injected with luciferin, euthanized, and organs (heart, lungs, liver, spleen, kidneys, tumor) removed. Tissues were imaged *ex vivo* for both **a.** bioluminescence to identify luciferase expressing tumor cells and **b.** fluorescence to visualize siRNA biodistribution. **c.** Tumor tissue was weighed, homogenized in PBS and pelleted. Cy5.5 fluorescence intensity in the supernatant was measured at 675 nm excitation and 694 nm emission wavelength. The picomolar amount of Cy5.5 per gram in each tissue was determined by relating the measured fluorescence to a standard curve generated by known quantities of Cy5.5 siRNA.

Ovarian cancer spreads locally within the abdominal cavity leading to tumor studding of peritoneal surfaces, blockage of lymphatic drainage and development of ascites. We used an intraperitoneal xenograft mouse model to recapitulate the typical clinical presentation in patients. Since assessment of intraperitoneal tumor burden by palpation is unreliable, we utilized the luciferase-expressing SKOV3 clone EA8 to permit visualization of tumors *in vivo* with bioluminescence imaging. Mice bearing SKOV3 EA8 intraperitoneal ovarian tumors were imaged after luciferin injection by the IVIS Xenogen imaging system and separated into treatment groups with similar tumor burden as measured by average bioluminescence intensity. Mice were then injected with siRNA labeled with the near-infrared dye Cy5.5. Mice were sacrificed and organs were removed and imaged in an *ex vivo* fashion. We observed enhanced *in vivo* localization of siRNA delivered by trastuzumab-directed polymer to intraperitoneal ovarian tumors. Combined bioluminescence and fluorescence imaging after 24 hours revealed the accumulation of Cy5.5 siRNA in the kidneys whereas Cy5.5 siRNA administered within trastuzumab-targeted nanoparticles was still present in tumors at this timepoint (Figure 5a, 5b). Quantification of Cy5.5 was accomplished by homogenizing tissue and measuring the amount of Cy5.5 fluorescence relative to a standard curve generated by a known quantity of Cy5.5 labeled siRNA. This revealed a marked accumulation of Cy5.5 in tumors removed from mice treated with Trast-SA bearing siRNA nanoparticles compared to free siRNA. Tumors from mice treated with Trast-SA targeted nanoparticles contained an average of 228 picomoles of Cy5.5 per gram of tumor tissue compared to 11 picomoles in tumors from mice treated with non-targeted siRNA, p=0.005 (Figure 5c). Comparison of the amount of Cy5.5 in tumor and kidney showed an average tumor to kidney ratio of 0.6 versus 50 (p=0.04) in mice treated with non-targeted siRNA or Trast-SA targeted siRNA respectively (data not shown). The average tumor to liver ratio of Cy5.5 was 1.8 versus 39 (p=0.002) in mice treated with non-targeted siRNA or Trast-SA targeted siRNA, respectively (data not shown).

### Effective suppression of target genes in intraperitoneal ovarian tumors

**Figure 6:**
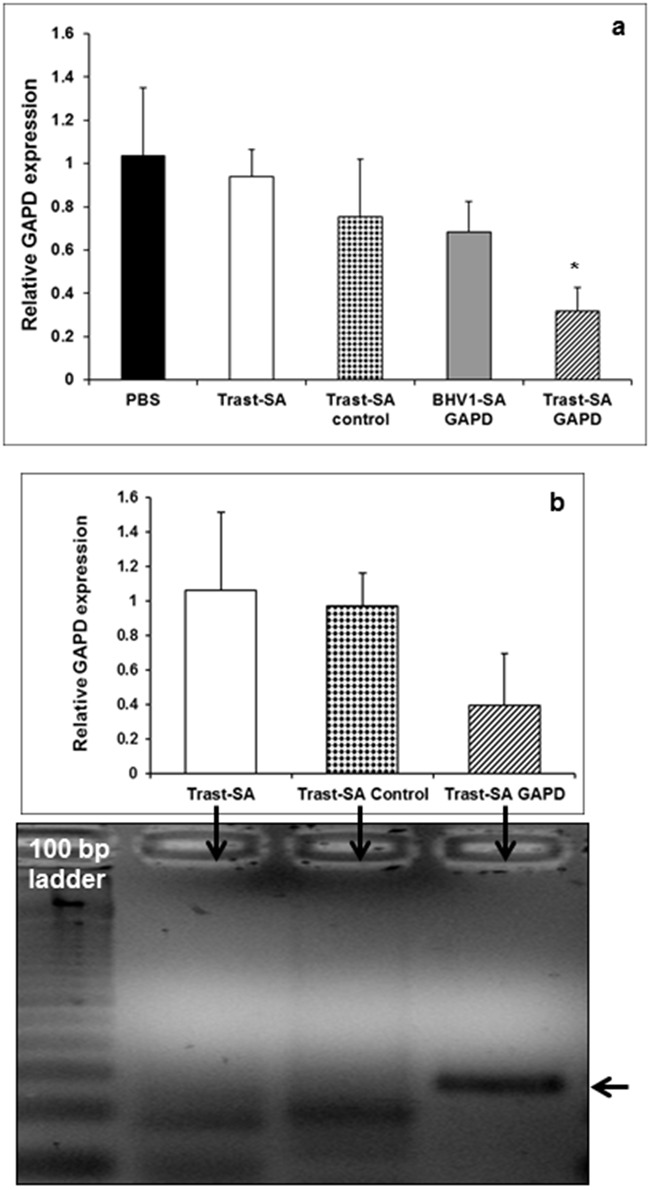
RNAi mediated target gene silencing within intraperitoneal ovarian cancer tumors after HER2 antibody directed siRNA delivery Athymic mice bearing intraperitoneal SKOV3 EA8 tumors verified by bioluminescence imaging were treated with Trast-SA or BHV1-SA conjugate bearing nanocarriers containing negative control or *GAPD* siRNA, Trast-SA alone or PBS at doses of **a.** 4 mg/kg (n=5 mice per group) or **b, c.** 2 mg/kg siRNA (n=3 mice per group) for two doses administered 24 hours apart. Mice were sacrificed 72 hours after the second dose and tumors collected for (a, b) qRT-PCR analysis and (c) detection of the 281 base pair *GAPD* mRNA cleavage product by 5′RLM-RACE assay. Error bars represent the standard deviation for qRT-PCR analyses. Asterisk indicates a significant difference (p<0.01) using the Student *t*-Test with one-tailed distribution between Trast-SA *GAPD* and BHV1-SA *GAPD*.

Functional delivery of *GAPD* siRNA to tumors *in vivo* was investigated. Mice bearing intraperitoneal SKOV3 EA8 tumors were divided into groups with a similar average tumor bioluminescence and treated with the following: PBS, Trast-SA alone, BHV1-SA targeted nanoparticles containing *GAPD* siRNA or Trast-SA targeted nanoparticles containing either control or *GAPD* siRNA. Mice were treated daily for two consecutive days at a dose of 4 mg/kg siRNA, then sacrificed 72 hours after the last dose. Intraperitoneal tumors were excised and RNA extracted. Reduction of *GAPD* gene expression by 70% with Trast-SA targeted nanoparticles containing *GAPD* siRNA was demonstrated via qRT-PCR compared to a 25% reduction with BHV1-SA non-targeted nanoparticles (Figure 6a). Additional mice were treated on the same schedule but at a dose of 2 mg/kg siRNA (Figures 6b, 6c). The 281 base pair predicted *GAPD* mRNA cleavage product was detected using the 5′RLM-RACE assay in ovarian tumor tissue derived from mice treated with Trast-SA bearing nanoparticles containing *GAPD* siRNA but not in mice treated with Trast-SA nanoparticles containing negative control siRNA or Trast-SA alone thus verifying RNAi-mediated *GAPD* gene suppression (Figure 6c). No induction of TLR3-activated immune response genes (*OAS1* and *STAT1*) was detected (Supplementary Figure S4a, 4b). A minor three-fold increase in expression of mouse *Ifit1* (interferon-induced protein with tetratricopeptide repeats-1), another marker of immune response, was detected in the spleens of mice treated with siRNA containing nanoparticles bearing either Trast-SA or BHV1-SA (Supplementary Figure S4). No marked changes in pancreatic, hepatic, or renal function, nor elevation in creatinine kinase or glucose were detected between treatment groups (data not shown).

## DISCUSSION

Effective intracellular delivery to target cells *in vivo* is undoubtedly the most difficult hurdle to overcome in the development of oligonucleotide therapeutics. Early phase clinical trials testing therapeutic siRNA in humans have shown favorable results, but it is still uncertain which delivery vehicle will ultimately prove to be the most effective for clinical application. We present encouraging preclinical data testing a modular antibody-directed biotinylated polymer carrier that can be easily adapted to different tumor types by varying the combination of antibody conjugate and target siRNA sequence.

We have demonstrated that this antibody-directed carrier can deliver functionally active siRNA to both ovarian and breast cancer cells overexpressing HER2. Two internalizing HER2 antibody conjugates (trastuzumab and 10H8) that recognize separate epitopes effectively facilitated intracellular delivery of siRNA. Non-specific limited uptake of particles bearing non-targeting BHV1-SA was observed and may be largely due to macropinocytosis which can be upregulated in cancer cells. [24] Blocking HER2 receptors with free Trast-SA conjugate prior to treatment with trastuzumab-bearing nanoparticles reduced the level of suppression to that seen with non-targeting BHV1-SA, supporting the presence of an alternate pathway of uptake independent of HER2 receptor binding. The residual cationic charge on nanoparticles can promote non-specific association with negatively charged components on the cell surface membrane. Charge neutral carriers may reduce spontaneous non-targeted cellular uptake and our group is exploring alternative non-cationic carrier chemistries that utilize direct covalent disulfide linkage of siRNA to polymer rather than electrostatic interaction. [25] These neutral carriers are effective *in vitro* but require a higher siRNA concentration and longer continuous incubation time to achieve a level of gene suppression comparable to the antibody-targeted cationic diblock polymer used in the studies described in this report. Incorporation of an internalizing antibody or ligand moiety to the neutral siRNA carrier may counteract any loss of cellular uptake due to diminished charge interaction and improve the specificity of delivery and reduce the *in vivo* toxicity that can be observed with cationic carriers. Efforts to test this hypothesis are ongoing.

Verification of the intended mechanism of action is important in evaluating the efficacy of a therapeutic carrier. Detection of the predicted target mRNA cleavage product by 5′RLM-RACE is the gold standard for confirmation of RNAi-mediated gene suppression. [26] We showed that the predicted mRNA cleavage products were detectable both by size on agarose gel electrophoresis and by sequencing of the PCR product using two different siRNAs designed against either *GAPD* or *BCL-XL* genes in cultured ovarian cancer cells. Furthermore, site specific cleavage of target mRNA was detected in mouse xenograft ovarian tumor tissue (Figure 6c) supporting the functional *in vivo* activity of siRNA delivered by HER2-targeted polymer carrier. Because activation of TLR3 by polymer-mediated siRNA delivery can lead to unintended effects from activation of the innate immune response, induction of *OAS1* and *STAT1* genes was assessed in tumors and found not to be upregulated *in vivo*. However, mouse *Ifit1* gene expression was increased approximately 3 to 4-fold in mouse spleen after treatment with Trast-SA or BHV1-SA targeted nanoparticles suggesting modest immune response activation. The significance of this low level of *Ifit1*upregulation is uncertain. Notably, siRNA treated mice exhibited no difference in behavior or appearance than mice receiving PBS alone. In comparison, a lipid-based carrier has been shown to induce a 247-fold increase of *Ifit1* mRNA expression and detectable serum interferon alpha levels in mice treated with 2 mg/kg unmodified siRNA compared to PBS treatment. [27] Mitigation of the immune response may be achieved by use of 2′O-methyl modification of siRNA sequences [27] or pretreatment with dexamethasone. [28]

In conclusion, we have demonstrated the successful *in vitro* and *in vivo* antibody-targeted functional delivery of siRNA to HER2-overexpressing cancer cells. RNAi-mediated cleavage of target mRNA was verified as the mechanism of gene suppression. Antibody-targeted nanoparticle delivery of siRNA incorporating endosomolytic polymer carriers may be a useful strategy for cancer therapy. Similar targeted carriers to deliver small molecule drugs or peptides may also be promising. Future directions include testing therapeutic siRNA sequences both as monotherapy and in combination with chemotherapy with *in vivo* cancer models. Evaluation of other internalizing receptors expressed on a higher proportion of ovarian tumors than HER2 such as mesothelin is also of interest. [29]

## MATERIALS AND METHODS

### Cell lines and media

The SKOV3, SKBR3 and BT-474 cell lines were obtained from the American Type Culture Collection (ATCC; Manassas, VA). Authentication of the cell line was assured by the provider. All three of these cell lines have *HER2* gene amplification and overexpression. Multiple aliquots were frozen from cells continuously passaged no longer than 3 months after initial receipt. Cells for experiments were passaged continuously for no longer than 3 months before being discarded and fresh aliquots resuscitated. The SKOV3 luciferase-expressing clone EA8 was generated by retroviral transduction of SKOV3 cells with a retrovirus encoding the firefly luciferase-Thy1.1-Neo construct [30] followed by flow cytometric sorting for Thy1.1 expression and selection of Thy1.1 expressing cells with G418. SKOV3 EA8 was subsequently cloned by limiting dilution. All cell culture reagents were obtained from Invitrogen (Carlsbad, CA) except for fetal bovine serum (FBS) obtained from Thermo Scientific Hyclone (Logan, UT). SKOV3, SKOV3 EA8 and SKBR3 cells were grown in RPMI containing 10% fetal bovine serum, 100 IU/ml penicillin, 100 mcg/ml streptomycin, and 2 mM L-glutamine with the addition of 800 mcg/ml G418 for the SKOV3 EA8 cells. BT-474 cell lines were grown in Hybri-Care Medium (ATCC) with 10% FBS. All cells were maintained at 37°C in 95% air/5% CO_2_ incubator.

### Polymer and antibody streptavidin conjugates

Polymer was synthesized as previously described.[19] BHV1 (IgG1 anti-bovine herpes virus-1 antibody) was produced from a hybridoma obtained from ATCC and purified from ascites fluid over a HiTrap Protein G HP column (GE Healthcare; Piscataway, NJ). Trastuzumab used for experiments was clinical grade (Genentech; San Francisco, CA). 10H8 (an internalizing antibody to HER2) was produced in the laboratory of Michael F. Press (University of Southern California) as published. [31] Streptavidin conjugations to trastuzumab, BHV1 and 10H8 antibodies were performed by Donald K. Hamlin (University of Washington) using previously published techniques. [32, 33]

### Polymeric micelle formation and transfection

Polymeric micelles were reconstituted from lyophilized polymer to a concentration of 1 mg/ml or 10 mg/ml in sterile PBS for *in vitro* or *in vivo* experiments respectively as previously described. [19] Polymer was added to siRNA at a polymer:siRNA molar ratio of 3:1 and incubated for 30 minutes at room temperature. The mAb-SA conjugates were added to polyplexes at 1:1 conjugate to available biotin molar ratios. Dynamic light scattering measurements of the polymeric micelles and siRNA complexes (N/P ratio = 3) in 150 mM PBS at pH 7.4 yielded hydrodynamic diameters of 34 nm and 37 nm respectively. The surface charge of the siRNA-polymer complexes (N/P ratio = 3), based on ζ-potential measurements was found to be 4 mV, which suggests that particles are slightly positive under these conditions.

Cells were plated at a cell density of 50,000/well in triplicate on the day prior to treatment. Cells were treated with polyplexes for 2 hours in medium containing 5% FBS followed by a medium change to 10% FBS. Cells were harvested at 48 or 72 hours post-treatment for RNA and protein extraction respectively. The siRNA sequences were as follows: glyceraldehyde-3-phosphate dehydrogenase (*GAPD*) (sense strand 5′-GGU CGGAGUCAACGGAUUUTT-3′; Integrated DNA Technologies; Coralville, IA); *STAT3* (sense strand 5′-GCCUCUCUG CAGAAUUCAATT-3′, Integrated DNA Technologies); *BCL-XL* (sense strand 5′-GCUGGA GUCAGUUUAGAUGATT-3′; siRNA ID# s1921; sense strand 5′-GGAACUCUAUGGGAACAAUTT-3′; siRNA ID# s1922, Ambion); Ambion *In Vivo GAPDH* siRNA Cat#4457291; Ambion *In Vivo* Bcl2L1 Cat#4457308; Silencer Negative Control #1 siRNA (Catalog AM4611; Ambion).

### Flow cytometry

Cells were plated at 200,000/well in 12-well plates the day prior to treatment. Polyplexes were formed as above using AlexaFluor 647-labeled AllStars Negative Control siRNA (#1027287; Invitrogen) and added to cells in media containing 5% FBS at a final concentration of 20 nM of siRNA. After incubation at 37°C to allow internalization, cells were trypsinized and acid washed to remove residual surface bound nanoparticles and intracellular fluorescence measured on a BD FACS Canto flow cytometer with untreated cells set as a background reference as previously described. [19]

### Fluorescence microscopy

SKOV3 EA8 cells were plated at 50,000/well in 2-well chamber slides and allowed to adhere overnight. Polyplexes were formed as above using AlexaFluor 647-labeled AllStars Negative Control siRNA (#1027287; Invitrogen) and added to cells in media containing 5% FBS at a final concentration of 25 nM of siRNA. After incubation at 37°C for 2 hours, cells were rinsed to remove unbound nanoparticles and fresh medium added. After 24 hours, cells were rinsed three times in PBS, fixed with 10% neutral buffered formalin for 15 minutes, and rinsed again in PBS prior to being coverslipped with Prolong Gold antifade reagent with DAPI (Invitrogen). Random fields were imaged at 60x (oil) with a DeltaVision Elite wide-field deconvolution microscope (Applied Precision, Issaquah, WA) fitted with a Photometrics HQ scientific grade cooled CCD camera and an Olympus 60×/1.42 Plan Apochromatic objective. Acquisition settings were constant for the AlexaFluor 647 signal. Three-dimensional data sets consisting of optical sections at 0.2 micron spacing were collected with the manufacturer's SoftWoRx software, and deconvolved using a constrained iterative algorithm. Data sets were processed to normalize intensities to the same scale using SoftWoRx software. Merging the data sets with their respective differential interference contrast (DIC) images was done with public domain software ImageJ.

### Quantitative RT-PCR

RNA was isolated using the RNeasy mini kit (Qiagen; Valencia, CA) and reverse transcribed using TaqMan Reverse Transcription reagents (Applied Biosystems; Carlsbad, CA). PCR reactions were run in duplicate using an ABI Prism 7900HT real-time PCR instrument. Primers with FAM and VIC-labeled compatible probes for multiplex PCR were obtained from Applied Biosystems: *GAPD* (Catalog#4352934E), *peptidylprolyl isomerase A* (*PPIA*) (Catalog#4326316E), *BCL-XL* (Hs00236329_m1), *STAT3* (Hs00374280_m1), *STAT1* (Hs01013996_m1), *OAS1* (Hs00973637_m1), mouse *Ifit1*(Mm00515153_m1). Relative quantification of gene expression was based on the reference value from the untreated control. [34]

### 5′-RLM-RACE and sequencing

5′ RLM-RACE was performed using the GeneRacer Kit (Invitrogen) with some modification as previously described. [35] Briefly, 100 ng total RNA was directly ligated to 250 ng GeneRacer RNAOligo with T4 ligase. After phenol/chloroform extraction and ethanol precipitation, cDNA was synthesized using random primers. From this reaction, 1 μl was used for first round 5′RACE reaction using the GeneRacer 5′primer and gene-specific primers (*GAPD*-specific reverse primer, 5′-CCTGCAAATGAGCCCCAGCCTTCTC-3′or *BCL-XL*- specific reverse primer, 5′-TCTACGCTTTCCACGCA CAGTGCCC-3′) with the following cycling conditions: 1 cycle of 94°C for 2 minutes, then 5 cycles of 94°C for 30 seconds and 72°C for 1 minute, then 5 cycles of 94°C for 30 seconds and 70°C for 1 minute, then 20 cycles of 94°C for 30 seconds, and 68°C for 1 minute. Second-round 5′ RACE reaction was then performed using 1 μl of the first-round reaction and internal GeneRacer 5′ nested and gene-specific primers (*GAPD*-specific nested, 5′-CGCCAGCATCGCCCCACTTGATTTT-3′or *BCL-XL*-specific nested, 5′-GCTGTCCCTGGGGTGA TGTGGAGCT-3′) using the above cycling conditions except for an extension time of 15 seconds and 25 cycles. PCR were performed using an Eppendorf Mastercycler thermocycler. PCR products were run on a 3% agarose gel containing ethidium bromide then excised and extracted using a QIAquick Gel Extraction kit (Qiagen). Sequencing was performed using the ABI BigDye Terminator v3.1 Cycle Sequencing kit and subsequently analyzed on an ABI-3730xl DNA Analyzer (Applied Biosystems) per manufacturer′s protocol.

### Immunoblotting

Protein gel electrophoresis was performed by loading 5 μg of cell lysate per lane on a 4-12% Bis-Tris NuPAGE gel (Invitrogen) followed by transfer to a PVDF membrane (Invitrogen). The membrane was blocked with 5% Milk/TBS (0.1%Tween20), prior to overnight incubation with primary antibody incubation at 4°C. Secondary antibody probing (Polyclonal Goat Anti-Rabbit Immunoglobulins/HRP; Dako; Carpinteria, CA) was done for 1 hour at room temperature. Results were visualized using Amersham ECL Western Blotting Detection Reagents (GE Healthcare; Pittsburg, PA) and Amersham Hyperfilm ECL (GE Healthcare). Reprobing to verify loading control was done after rinsing the membrane and reblocking before subsequent antibody probing. BCL-XL (clone 54H6), GAPD (clone 14C10), and beta-actin (clone 13E5) antibodies for immunoblotting were obtained from Cell Signaling Technologies (Danvers, MA).

### *In vitro* toxicity analysis

Cells were plated in quadruplicate at a cell density of 5,000 cells/well in white 96-well plates on the day prior to treatment. Cells were treated with polyplexes for 2 hours then harvested 48 hours later and assayed using the CellTiter-Glo Luminescent Cell Viability Assay (Promega) per manufacturer's instructions. Microplates were read on a Centro LB 960 microplate luminometer (Berthold Technologies).

### GAPD protein assay

GAPD enzyme activity was quantitated using the KDAlert GAPDH assay kit (Ambion) per manufacturer's recommendations. Cells were treated in quadruplicate then lysed 72 hours later. Lysate was transferred to a 96-well plate and KDAlert master mix added. The fluorescence intensity was measured over 4 minutes using an excitation wavelength of 560 nm and emission wavelength of 590 nm.

### Xenograft experiments

5-6 week old female athmyic *nu/nu* mice weighing approximately 20 grams were obtained from Harlan Laboratories. Animals were housed in specific pathogen-free housing. All experiments were performed with the approval of the Fred Hutchinson Cancer Research Center Institutional Animal Care and Use Committee. 10 million SKOV3 luciferase-expressing cells suspended in sterile PBS (Gibco) were injected intraperitoneally. For imaging studies, mice were injected intraperitoneally with firefly D-luciferin (Biosynth International, Inc; Itasca, IL) at 150 micrograms per gram of body weight and tumor establishment was verified by *in vivo* bioluminescence imaging using the Xenogen Spectrum system (Perkin Elmer; Waltham, MA). Mice were separated into treatment groups with similar average bioluminescence intensity and then injected intraperitoneally with Cy5.5-labeled siRNA alone, a trastuzumab-targeted polyplex containing Cy5.5-labeled siRNA, or sterile PBS alone. After 24 hours, mice were euthanized via carbon dioxide inhalation. Organs were removed and imaged in an *ex vivo* fashion. Kidney, liver, and tumor nodules were flash frozen for further analysis. For functional siRNA delivery experiments, mice were injected intraperitoneally with 2 or 4 mg/kg siRNA once daily for two days then euthanized via carbon dioxide inhalation 72 hours after the second dose. Blood for laboratory toxicity analysis was harvested via cardiac puncture. Tumor and spleen were placed in RNAlater (Qiagen) for RNA extraction for qRT-PCR and 5′RACE analysis (tumor only).

### RNA extraction and quantitation from tissue

RNA was extracted from mouse tissues as previously described. [36] Briefly, samples were homogenized in guanidine thiocyanate lysis buffer with the FastPrep-24 instrument (MP Biomedicals; Solon, OH) and Lysing Matrix D tubes (MP Biomedicals). Samples were immediately centrifuged at 4°C, extracted with phenol-chloroform and RNA precipitated with isopropanol and sodium acetate (Ambion) overnight at −20°C. Further RNA purification was performed using the RNeasy kit (Qiagen; Valencia, CA) per manufacturer's instructions after adding 350 μl RNEasy RLT buffer and 100 μl 100% ethanol to the resuspended RNA pellet. Residual DNA contamination was removed with the Turbo DNA-Free kit (Ambion). The supernatants were collected and quantitated via UV-Vis for concentration analysis.

### Biodistribution

Quantification of Cy5.5-labeled siRNA in tissues was performed as previously described. [37] Briefly, tissue was homogenized in PBS with the FastPrep-24 instrument and Lysing Matrix D tubes. Homogenates were centrifuged to remove insoluble tissue debris. The fluorescence intensity of the supernatant was analyzed using a Synergy H4 microplate reader (BioTek; Winooski, VT) at 675 and 694 nm of excitation and emission wavelength, respectively. The moles of siRNA per gram of tissue was determined by using a standard curve generated by diluting 0.5 μmoles of Cy5.5-labeled *GAPD* siRNA 1:2 in PBS in succession six times. Tissue samples were diluted 1:2 in PBS before being read in duplicate along with the standards and a blank PBS sample.

## SUPPLEMENTARY FIGURES


