# Supplementary material for: Antibody targeting facilitates effective intratumoral siRNA nanoparticle delivery to HER2-overexpressing cancer cells
Source: Oncotarget. 2016 Jan 30;7(8):9561–75. doi: 10.18632/oncotarget.7076 (PMC4891060; doi:10.18632/oncotarget.7076)
Supplement: Supplementary file 1 [file oncotarget-07-9561-s001.pdf]

SUPPLEMENTARY FIGURES

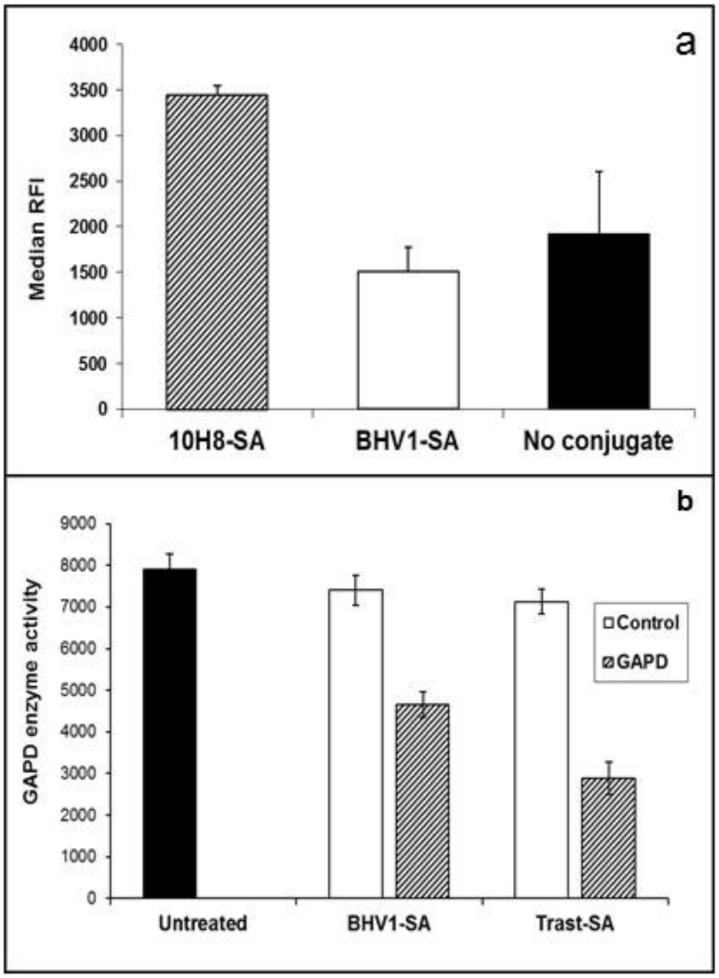

Supplementary Figure S1: Effective siRNA uptake and gene suppression mediated by an alternative HER2 antibody 10H8. a. SKOV3 cells were treated with nanoparticles bearing BHV1-SA or 10H8-SA conjugates or naked nanoparticles containing AlexaFluor 647 labeled siRNA for 1.5 hours prior to harvest, acid rinse and flow cytometric analysis. Median relative fluorescence intensity (RFI) was measured. Error bars represent the standard deviation of replicate treatments. **b.** SKOV3 cells were treated with a 3 hour pulse of BHV1-SA, Trast-SA or 10H8-SA bearing nanoparticles containing 10 nM negative control or *GAPD* siRNA and assayed for GAPD enzyme activity 72 hours later.

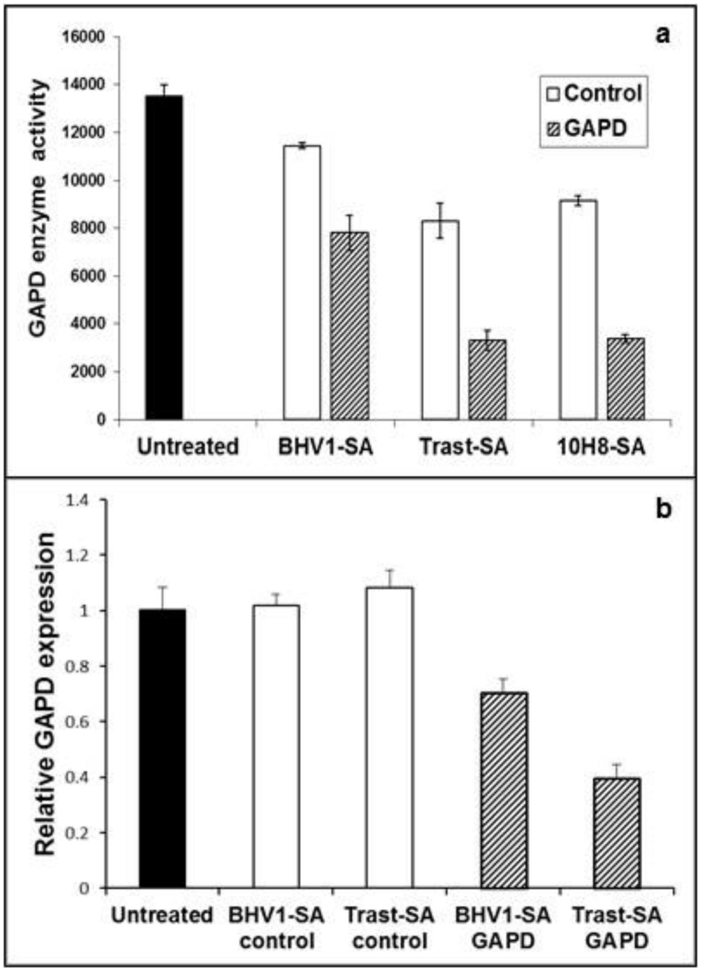

Supplementary Figure S2: Suppression of target genes in breast cancer cell lines SKBR3 and BT-474. a. SKBR3 cells were treated with a 3 hour pulse of BHV1-SA, Trast-SA or 10H8-SA bearing nanoparticles containing 15 nM negative control or *GAPD* siRNA and assayed for GAPD enzyme activity 72 hours later. Treatments were done in quadruplicate. Error bars represent standard deviation. **b.** BT-474 cells were treated in triplicate with a 2 hour pulse of BHV1-SA or Trast-SA bearing nanoparticles containing 25 nM siRNA then harvested and assayed for *GAPD* mRNA level 48 hours later. Error bars represent standard deviation.

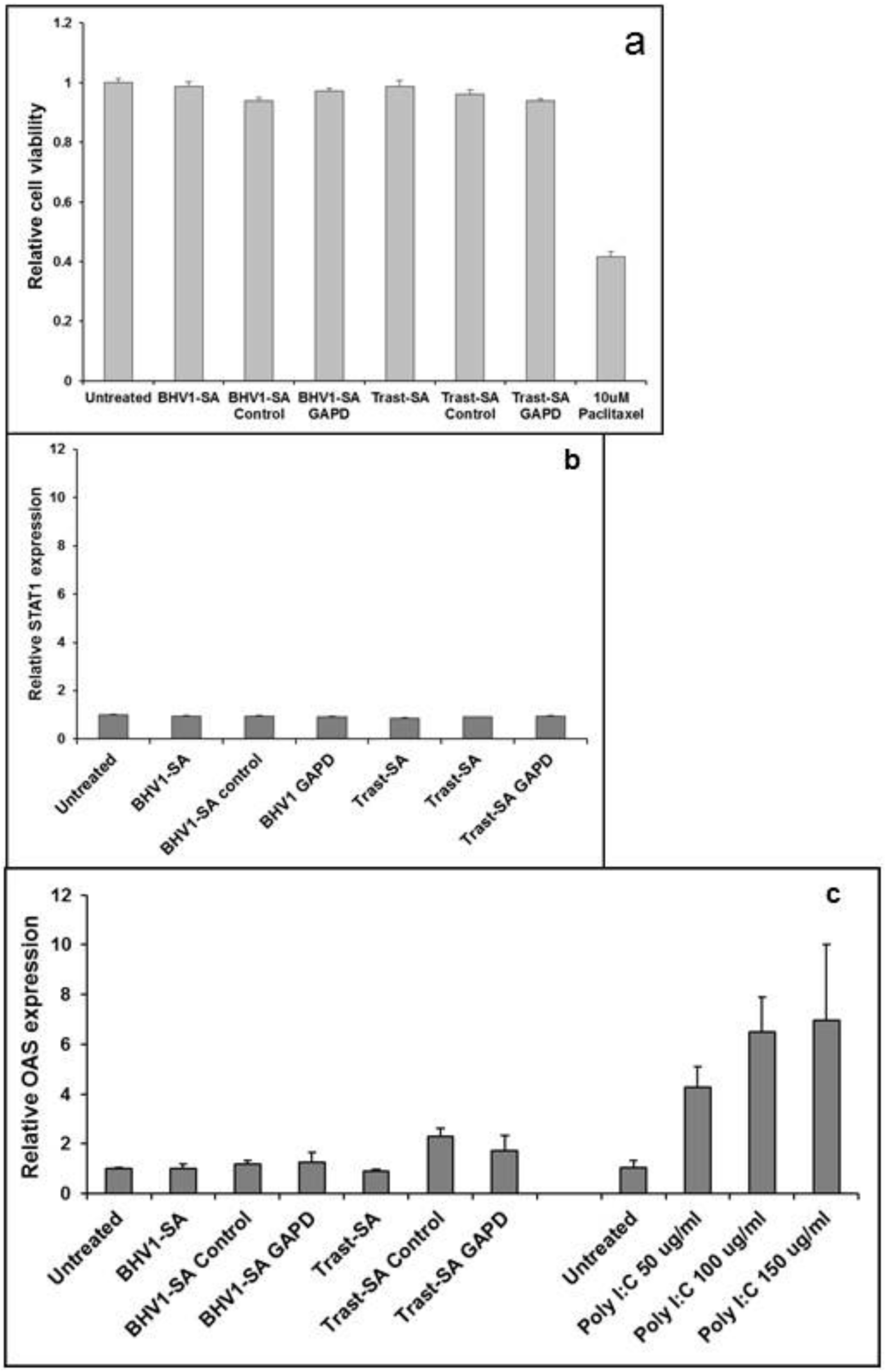

Supplementary Figure S3: Evaluation of *in vitro* toxicity and cytokine induction. a. SKOV3 cells were treated in quadruplicate with a 2 hour pulse of Trast-SA or BHV1-SA carriers containing 25 nM siRNA or antibody conjugates alone then assayed 48 hours later for viability using the CellTiter Glo Assay. Cells were treated with paclitaxel as a positive control for cytotoxic effect. Error bars represent standard deviation. **b, c.** SKOV3 cells were treated in triplicate with a 2 hour pulse of Trast-SA or BHV1-SA carriers containing 25 nM siRNA or antibody conjugates alone then assayed 48 hours later for induction of interferon activated immune response genes (b) STAT1 and (c) OAS1. Polyinosinic-polycytidylic acid (Poly I:C), a synthetic long double-stranded RNA, was used as a positive control for activation of TLR-3 mediated immune response. Error bars represent standard deviation.

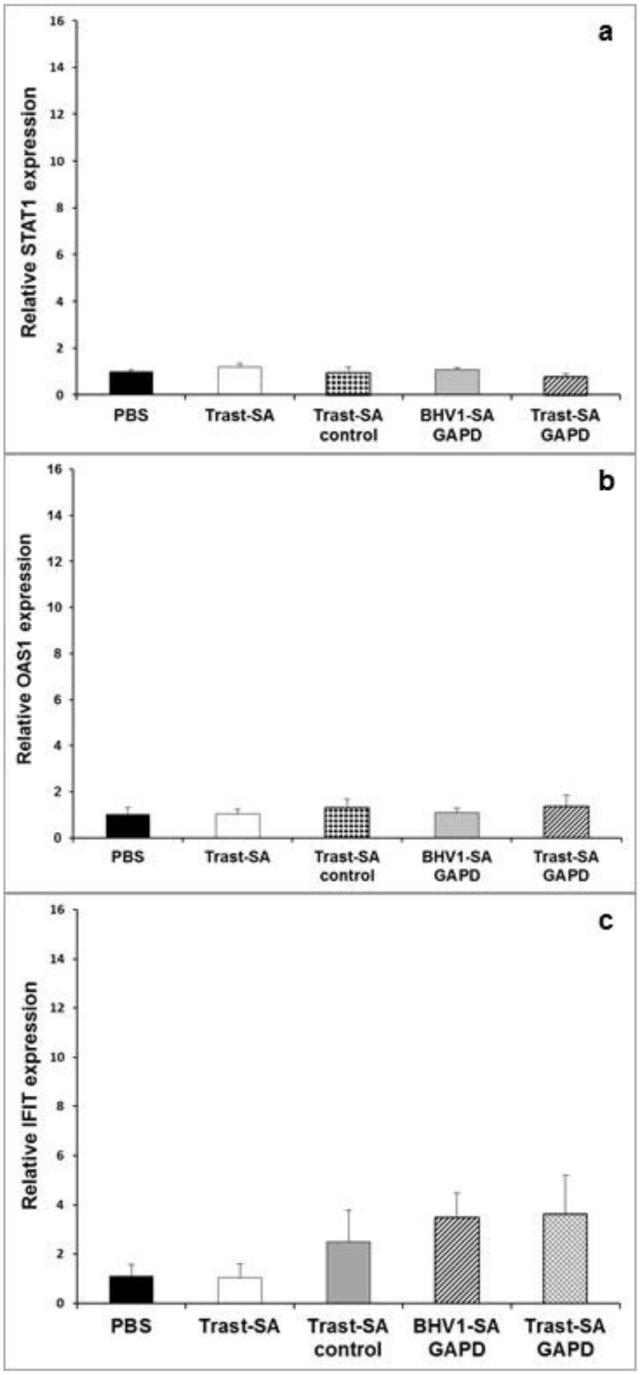

Supplementary Figure S4: Assessment of *in vivo* immune response. Tumor tissue and spleen were collected from mice bearing intraperitoneal ovarian tumors 72 hours after treatment with two daily doses of PBS, Trast-SA alone or nanoparticles bearing Trast-SA or BHV1-SA containing either control or *GAPD* siRNA at 4 mg/kg siRNA dose. RNA was extracted and the expression of human interferon response genes **a.** *STAT1* and **b.** *OAS1* in tumors and **c.** mouse *Ifit1*gene expression in spleen was measured by qRT-PCR. Error bars represent standard deviation.
